# Burden of autism spectrum disorder in Japan from 1992 to 2021 and its prediction until 2050: results from the GBD study

**DOI:** 10.3389/fpsyt.2025.1619085

**Published:** 2025-07-08

**Authors:** Jiabo Liu, Xiaoyan Xie, Yunxi Li, Yiqi Pu, Yan Li, Lingling Yang

**Affiliations:** ^1^ Second Clinical Medical College, Guangzhou University of Traditional Chinese Medicine, Guangzhou, Guangdong, China; ^2^ Department of Psychology and Sleep Medicine, Guangdong Provincial Hospital of Traditional Chinese Medicine, Guangzhou, Guangdong, China

**Keywords:** ASD, GBD, prevalence, DALYs, APC/AAPC

## Abstract

Autism Spectrum Disorder (ASD) is a neurodevelopmental condition characterized by persistent deficits in social communication and interaction, alongside restricted, repetitive patterns of behavior. This study aimed to analyze the temporal trends and project the future burden of ASD in Japan. Using Global Burden of Disease (GBD) 2021 data, we analyzed prevalence, disability-adjusted life years (DALYs), and age-standardized rates (ASR) (1992-2021) through age-period-cohort modeling, joinpoint regression, and autoregressive integrated moving average (ARIMA) forecasting. Age-standardized prevalence rate (ASPR) increased significantly (Average Annual Percentage Change [AAPC]=0.2744; 95%CI:0.2606-0.2882), with males disproportionately affected (male-to-female ratio 4:1). By 2050, crude prevalence is projected to decline 14.2%, while ASPR will rise 18.0%. Japan’s ASD burden exceeds global averages, necessitating targeted interventions across the lifespan. These findings highlight the increasing burden of ASD in Japan and underscore the urgent need for enhanced healthcare planning and resource allocation.

## 1 Introduction

Autism Spectrum Disorder (ASD), classified under the Diagnostic and Statistical Manual of Mental Disorders, Fifth Edition, Text Revision (DSM-5-TR) and the International Classification of Diseases, Eleventh Revision (ICD-11), constitutes a heterogeneous group of neurodevelopmental conditions characterized by persistent deficits in social communication and social interaction across multiple contexts, alongside restricted, repetitive patterns of behavior, interests, or activities (1, 2). The term “spectrum” reflects the substantial heterogeneity observed in symptom severity, functional impairment, and developmental trajectories. While core features typically emerge in early childhood, diagnosis can occur later, particularly in individuals with higher cognitive abilities or less pronounced symptoms (3).

The precise etiology of ASD remains incompletely elucidated; however, substantial evidence points to complex interactions between polygenic susceptibility and environmental influences (4). Globally, epidemiological studies report a significant increase in ASD prevalence estimates over recent decades. This observed rise is widely attributed to multiple factors: enhanced diagnostic sensitivity through refined criteria (DSM-5-TR, ICD-11) that better capture the spectrum’s breadth (1, 2, 5), heightened clinical and public awareness facilitating earlier recognition (6), and improved accessibility to diagnostic services and standardized screening tools (7). Current global prevalence estimates are approximately 1-2%, with a consistently reported male-to-female ratio near 4:1 (5, 8). Variations in prevalence across geographical regions and time periods may reflect differences in diagnostic practices, healthcare infrastructure, and cultural factors (5, 8, 9). Established risk factors include advanced parental age, familial aggregation suggestive of heritable components, prenatal complications, and preterm birth (10).

Within Japan, epidemiological trends mirror this global increase in ASD identification (11). Recent studies indicate prevalence rates in Japanese children and adults converging with estimates from Western nations (11, 12). Specific socio-cultural and familial environmental contexts within Japan may influence the recognition and expression of ASD traits, warranting further investigation (13). The Japanese healthcare system has implemented various interventions, including early screening initiatives, Applied Behavior Analysis (ABA), Early Intensive Behavioral Intervention (EIBI), speech-language therapy, social skills training, and vocational support programs (14). Concurrently, policy measures provide financial assistance, professional counseling, and educational resources for affected individuals and families (15). Despite these advancements, challenges persist, including societal stigma impacting families (16), and the need for enhanced employment opportunities and social inclusion supports for adults with ASD (17). Japan contributes significantly to international ASD research, particularly in the domains of genetics and neuroimaging (18), with emerging investigations leveraging advanced methodologies such as deep learning and genomic analyses (19, 20).

Quantifying the population health impact of ASD is essential for public health planning and resource allocation. The Global Burden of Disease (GBD) study provides a rigorous, comparative framework for assessing the burden of diseases and injuries, including ASD, using standardized metrics such as prevalence, incidence, years lived with disability (YLDs), and disability-adjusted life years (DALYs) across time and geography (21). While prior GBD iterations (e.g., GBD 2019) have documented the global burden of ASD (21), and recent analyses (e.g., GBD 2021 Autism Spectrum Collaborators) have specifically explored its global epidemiology (22), comprehensive analyses focusing on the longitudinal trends and future projections of ASD burden within Japan using the GBD framework remain limited.

Therefore, this study aims to: (1) Analyze the burden of ASD in Japan – encompassing prevalence, incidence, YLDs, and DALYs – from 1992 to 2021 utilizing data from the Global Burden of Disease Study; (2) Identify key factors associated with observed trends; and (3) Project the potential burden of ASD in Japan up to the year 2050. This analysis seeks to inform national health policies and service planning for individuals with ASD in Japan.

## 2 Materials and methods

### 2.1 Data source and study scope

This longitudinal analysis utilized data from the Global Burden of Disease Study 2021 (GBD 2021), coordinated by the Institute for Health Metrics and Evaluation (IHME). GBD 2021 provides comprehensive, standardized estimates of disease burden for 371 diseases and injuries across 204 countries and territories, including Japan and its 47 prefectures, from 1990 to 2021s (21, 22). Data were accessed via the IHME Global Health Data Exchange (GHDx) (https://vizhub.healthdata.org/gbd-results/). The study assessed the burden of ASD, defined according to GBD 2021 case definitions and mapped to ICD-10 codes (primarily F84) and DSM-5 criteria (1, 2, 21, 22). We extracted data for Japan (overall and by prefecture), the High-Income Asia Pacific region, High Socio-demographic Index (SDI) locations globally, and the Global aggregate for the period 1992–2021. Key outcome metrics extracted were: Prevalence: The total number of individuals living with ASD at a specific point in time (mid-year 2021 for point estimates; annual estimates for trends). Disability-Adjusted Life Years (DALYs): The sum of Years Lived with Disability (YLDs) and Years of Life Lost (YLLs) due to ASD. Given the low premature mortality associated with ASD, DALYs primarily reflect YLDs for this condition (21, 22). Age-Standardized Rates (ASRs): Rates adjusted to the GBD World Standard Population structure to enable comparison across populations with differing age distributions.

### 2.2 Key metrics and definitions

#### 2.2.1 Prevalence

Prevalence quantifies the proportion of a population living with ASD at a specific point in time. It is a core indicator of disease burden, reflecting the population-level impact of ASD and facilitating comparisons across groups, time, and geography (21, 22).

#### 2.2.2 Disability-adjusted life years

DALYs represent the total health loss associated with ASD, combining: Years Lived with Disability (YLDs): Calculated as prevalence multiplied by a disability weight specific to ASD, reflecting the severity of health loss associated with the condition (21, 22). Years of Life Lost (YLLs): Calculated as the number of deaths due to ASD multiplied by the standard life expectancy at the age of death. Due to the very low mortality directly attributable to ASD, YLLs contribute minimally to ASD DALYs (18, 21, 22).

#### 2.2.3 Age-standardized rates

To account for confounding by population age structure and enable valid comparisons, we utilized: Age-Standardized Prevalence Rate (ASPR): Prevalence per 100,000 population standardized to the GBD World Standard Population. Age-Standardized DALY Rate (ASDAR): DALYs per 100,000 population standardized to the GBD World Standard Population.

#### 2.2.4 Trend analysis metrics

Annual Percentage Change (APC): The estimated percentage change per year within a specific, homogeneous time segment identified by joinpoint regression. Average Annual Percentage Change (AAPC): A summary measure of the trend over the entire study period (1992–2021), calculated as a geometrically weighted average of the APCs from the joinpoint model (19–21).

#### 2.2.5 Socio-demographic index

SDI is a composite measure (scale: 0-1, where 0 represents the lowest theoretical level of development) of lag-distributed income per capita, average educational attainment in the population aged 15 and older, and the total fertility rate under age 25 (22). Locations are categorized into quintiles (e.g., High SDI represents the top 20% of locations globally based on SDI in a given year). Japan is classified within the High SDI quintile.

### 2.3 Analytical methods

#### 2.3.1 Descriptive analysis

We summarized crude and age-standardized estimates (Prevalence, DALYs, ASPR, ASDAR) by year, sex, age group, geographic level (Japan prefectures, Japan national, High-Income Asia Pacific, High SDI, Global), and SDI. Absolute numbers and rates with 95% uncertainty intervals (UIs) were reported.

#### 2.3.2 Temporal trend analysis (1992-2021)

Joinpoint Regression Analysis: We employed joinpoint regression (using the Joinpoint Regression Program, National Cancer Institute) to identify significant inflection points (joinpoints) in the temporal trends of ASPR and ASDAR (19–21). The optimal number of joinpoints (0 to N max) was determined using permutation tests (α=0.05). For each resulting linear segment, the Annual Percentage Change (APC) and its 95% confidence interval (CI) were calculated. The Average Annual Percentage Change (AAPC) over the entire period (1992-2021) was computed as a summary measure. Statistical significance was set at p<0.05. Pearson Correlation Analysis: The association between AAPC values for ASPR/ASDAR and baseline (1992) ASR levels or SDI values across Japanese prefectures was assessed using Pearson correlation coefficients (r) and linear regression.

#### 2.3.3 Decomposition analysis

To quantify the relative contributions of key drivers to changes in the absolute number of prevalent ASD cases and DALYs in Japan between 1992 and 2021, we performed a factor decomposition analysis (23). Changes were decomposed into contributions from. Population Growth: Change due solely to the increase in total population size. Population Aging: Change due to shifts in the age distribution of the population. Epidemiological Change: Change due to variations in age-specific prevalence or DALY rates (reflecting changes in risk, diagnosis, or disability weighting).

#### 2.3.4 Burden projection (2022-2050)

Future trends in ASPR and ASDAR for Japan were projected to 2050 using the Autoregressive Integrated Moving Average (ARIMA) model. Model selection (p, d, q parameters) was based on:

Examination of Autocorrelation Function (ACF) and Partial Autocorrelation Function (PACF) plots. Minimization of the Akaike Information Criterion (AIC) and Bayesian Information Criterion (BIC). Assessment of model residuals for white noise using the Ljung-Box test. The best-fitting ARIMA model for each outcome (ASPR, ASDAR) was identified using the auto.arima() function within the forecast package in R (24, 25). Model fit was validated by comparing predicted values for the final years of the observed data (e.g., 2015-2021) against actual GBD estimates. Projections are presented with 95% prediction intervals. The Mean Absolute Percentage Error (MAPE) was calculated for the validation period to assess forecast accuracy.

#### 2.3.5 Software

All statistical analyses, excluding joinpoint regression, were performed using R software (Version 4.3.1). Key packages included gbd (for data access/processing), forecast, ggplot2, and demography (for decomposition).

## 3 Result

### 3.1 Comparison of the different regional of ASD by prevalence and DALYs

**Figure 1:**
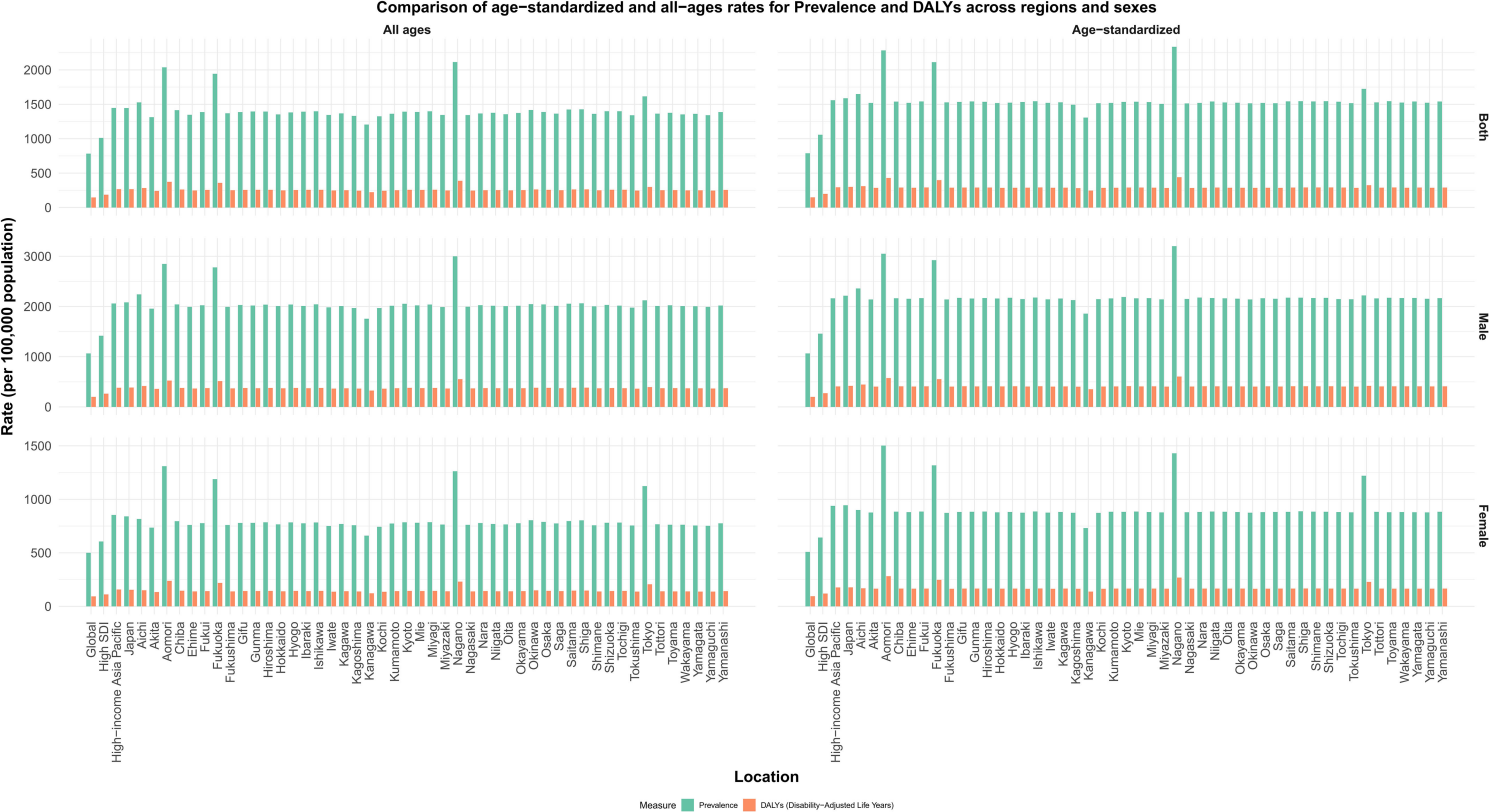
Comparison of age−standardized and all−ages rates for Prevalence and DALYs across regions and sexes.

**Figure 2:**
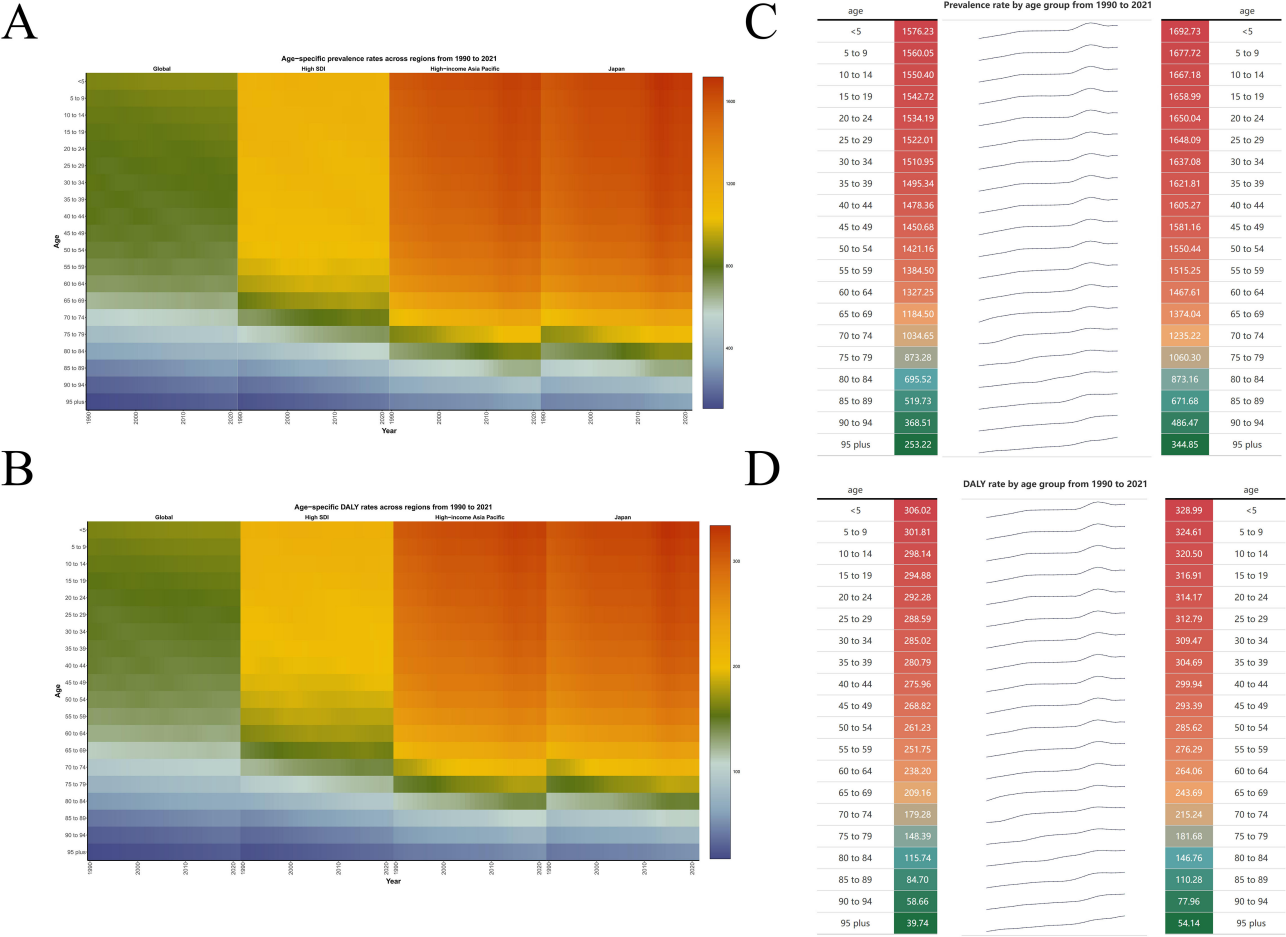
**(A, B)** ASR of prevalence and DALYs across regions from 1990 to 2021. **(C, D)** Prevalence and DALYs rate by age group from 1990 to 2021.

**Figure 3:**
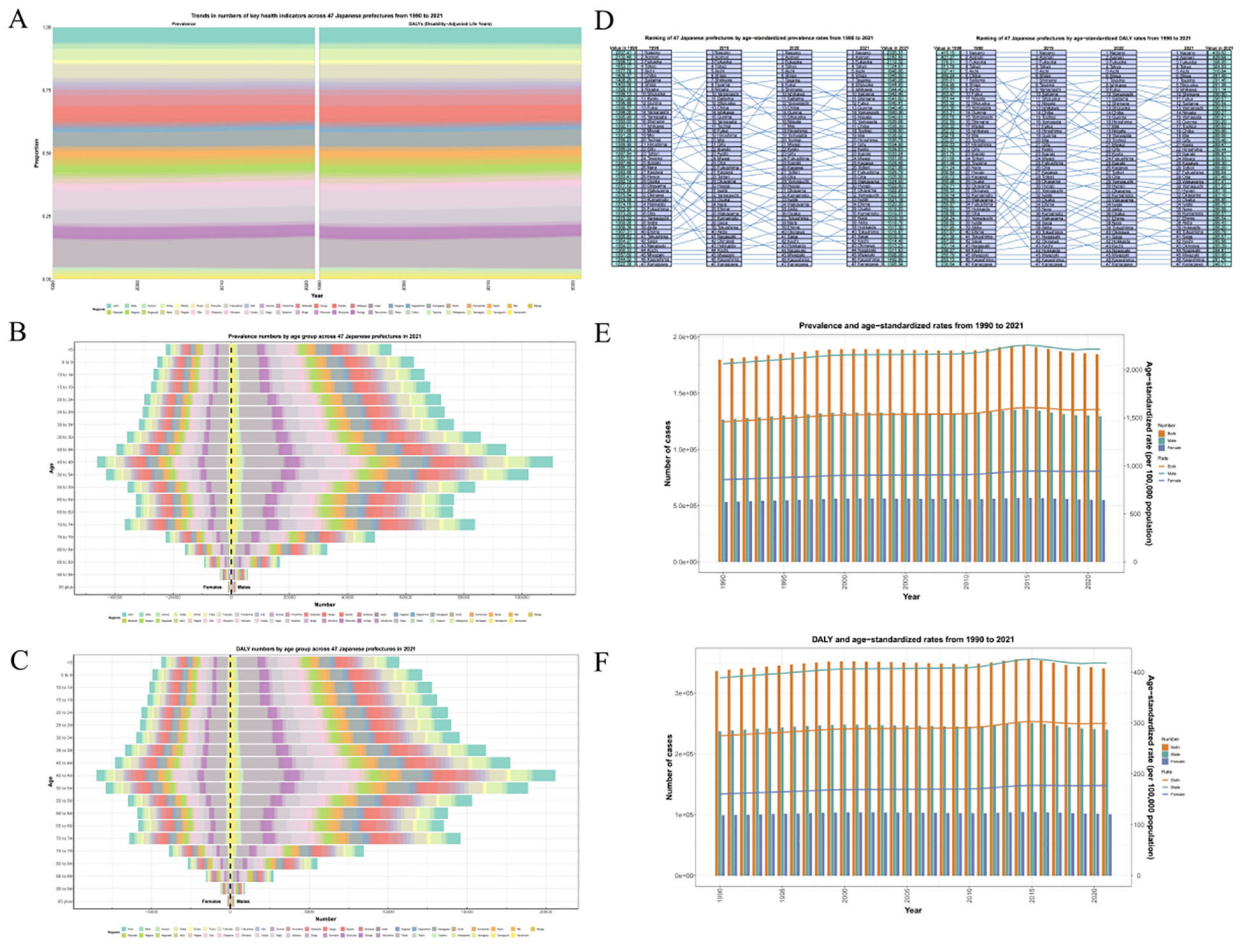
**(A)** Trends in numbers of key health indicators across 47 Japanese prefectures from 1990 to 2021. **(B, C)** Prevalence and DALYs numbers by age group across 47 Japanese prefectures in 2021. **(D)** Ranking of 47 Japanese prefectures by age−standardized prevalence and DALYs rates from 1990 to 2021. **(E, F)** Prevalence/DALYs and ASPR/ASDAR from 1990 to 2021.

The bar chart compares the burden of ASD in Japan’s 47 administrative regions with those in the global, high-SDI and high-income Asia-Pacific regions, showing the results at all ages and after age standardization. We found that prevalence and DALYs were significantly higher than global levels and high SDI regions in almost all administrative regions in Japan, while the high-income Asia-Pacific region was similar to the national burden of disease in Japan. Aomori, Fukuoka, Nagano, and Tokyo have a more prominent disease burden (**Figure 1**). Further deconstructing the data according to gender, we found that the prevalence rate and DALYs of males were higher than those of females by shown of heatmap (**Supplementary Figure S1**). Based on the data changes from 1992 to 2021, the prevalence and DALYs trends with time are demonstrated by using area charts and graphs. It can be found that prevalence and DALYs fluctuate over time, but the overall trend is increasing, and the disease group is becoming younger, and the disease burden is increasing in the younger age group (**Figure 2**). We divided Japanese ASD patients into 47 administrative regions and used a map to show the trend of ASD prevalence and DALYs proportion per capita over time in different administrative regions (**Figure 3A**). A pyramid chart showed the comparison of ASD prevalence and DALYs in different age groups in different administrative regions, which revealed that the peak age group for ASD was 45–49 years old, and a secondary peak age group was 70–74 years old (**Figures 3B, C**). We ranked the disease burden of the 47 administrative regions according to the time-varying trend and performed a paired comparison, which showed that the top four administrative regions, Nagano, Aomori, Fukuoka, and Tokyo, maintained their positions, while the rankings of the remaining 43 administrative regions changed over time (**Figure 3D**). Finally, we used a dual-axis analysis to show the trend relationship between prevalence and ASR, DALYs and ASR over time, which suggested that both prevalence and DALYs were associated with the time trend of ASR (**Figures 3E, F**).

### 3.2 Regional, gender, and age differences effect on trends in the burden of ASD

Joinpoint analysis revealed the change trend of disease burden of ASD under different variables (region, gender, age) from 1992 to 2021, and APC and AAPC were calculated for comparison.

**Figure 4:**
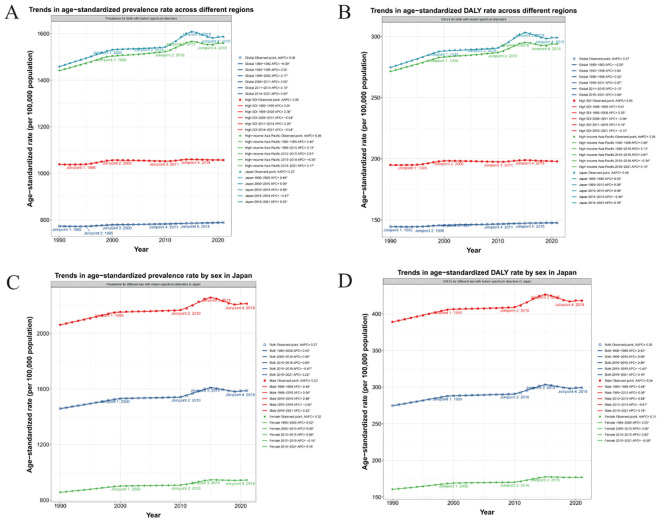
**(A, B)** Trends in ASPR and ASDAR across different regions with APC and AAPC. **(C, D)** Trends in ASPR and ASDAR rate by sex in Japan with APC and AAPC.

**Figure 5:**
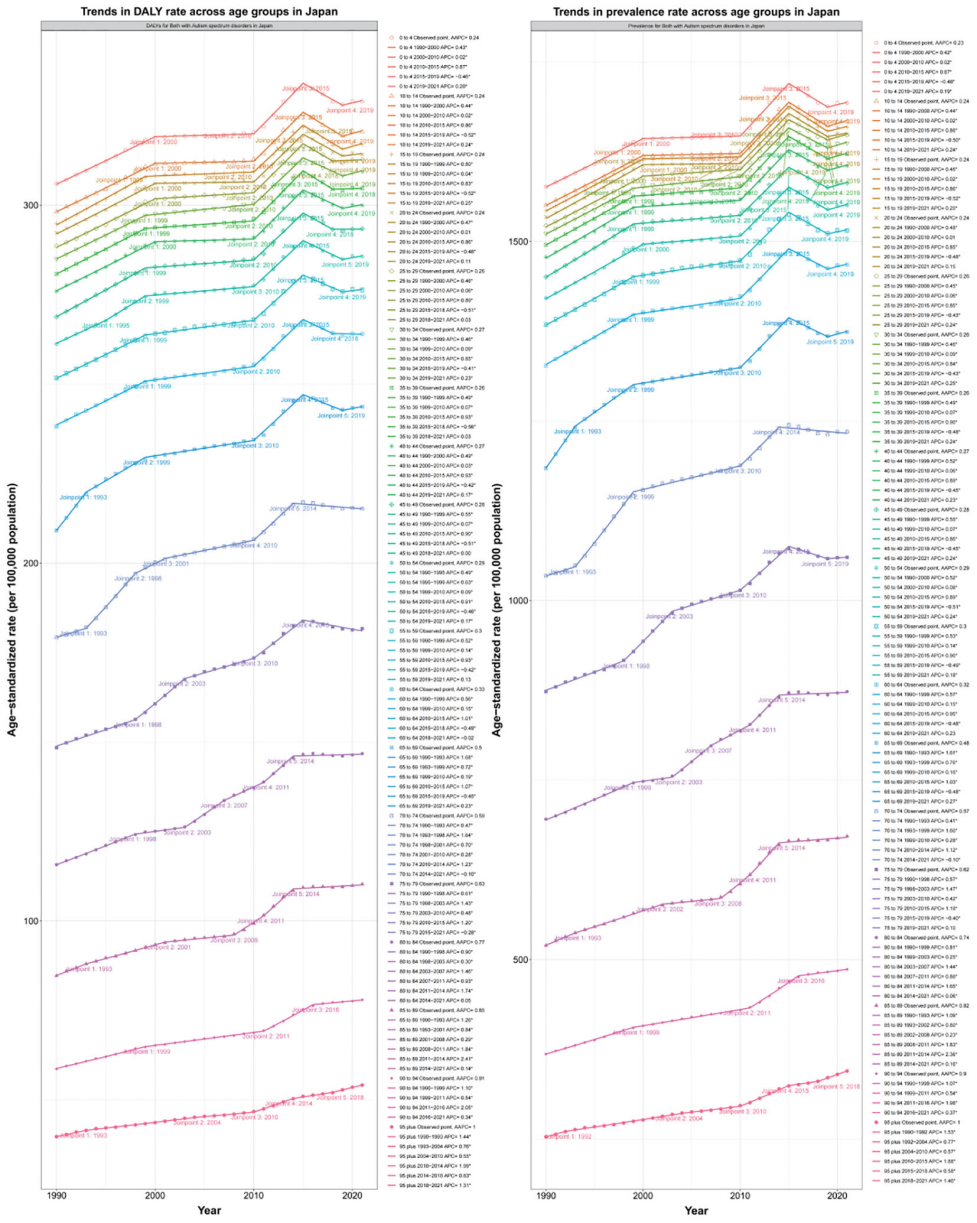
Trends in prevalence and DALY rate across age groups in Japan from 1990–2020 with APC and AAPC.

The results show that the highest ASPR APC period was 2010-2015, APC=0.89(95%CI:0.85, 0.94), and the lowest ASPR APC period was 2015-2019, APC=-0.47(95%CI: -0.54, -0.4). The highest ASDAR APC period was 2010-2015, APC=0.89(95%CI: 0.85-0.92), and the lowest ASDAR APC period was 2015-2019, APC=-0.45(95%CI: -0.5, -0.4). The national AAPC values of ASPR and ASDAR in Japan from 1992 to 2021 were 0.2744 (95%CI: 0.2606, 0.2882) and 0.2782 (95%CI: 0.2673, 0.2892), respectively. The results were 0.2342 (95%CI: 0.2215, 0.2469) and 0.2362 (95%CI: 0.2248, 0.2475) for males and 0.3177 (95%CI: 0.2978, 0.3375) and 0.3117 (95%CI: 0.2342, 0.2469) and 0.2362 (95%CI: 0.2475) for females. 0.2955, 0.328. The APC and AAPC values of different time stages, regions and genders can be referred to **Supplementary Tables S1, S2**. ASPR and ASDAR have increased year by year globally in the past 30 years, and the trend in Japan is close to the same as that in the Asia-Pacific high-income region (AAPC 0.27 vs 0.26), which is much higher than that in the global region and the region with high SDI (AAPC 0.27&0.26 vs 0.06&0.06). This result illustrates the high level of disease burden of ASD in Japan and its region (**Figures 4A, B**). After gender differentiation, it was found that the trend of disease burden indicators of different genders also increased year by year, but the ASPR and ASDAR of males were higher than those of females, indicating that males have always occupied a dominant position in the increasing ASD population in Japan (**Figures 4C, D**). After age differentiation, we found that on the basis of the increase of the overall disease burden index over time, ASPR and ASDAR also increased with the increase of age (**Figure 5**).

### 3.3 Age-period-cohort analysis reveals the disease burden difference in each administrative district throughout Japan from different age, period, and cohort

**Figure 6:**
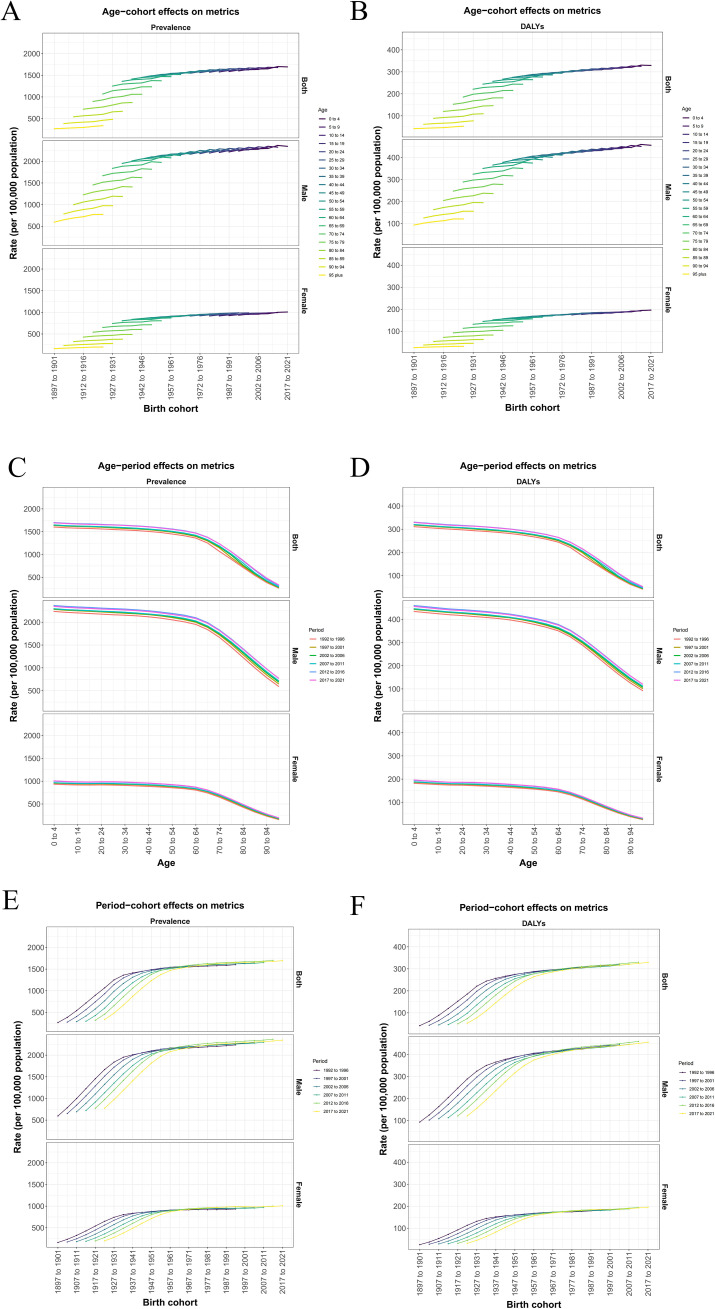
**(A, B)** Age−cohort effects on prevalence and DALYs metrics. **(C, D)** Age−period effects on prevalence and DALYs metrics. **(E, F)** Period-cohort effects on prevalence and DALYs metrics.

APC analysis decomposed the time dimension into age effect, period effect and cohort effect in order to unpack the independent influence of age, period and cohort. We calculated and plotted the age-period, time-cohort, age-cohort correlation curves with prevalence and DALYs in Japanese ASD patients by sex to determine the impact of different factors on disease burden. On this basis, APC analysis is conducted separately for the whole country and various administrative regions of Japan, and calibration is conducted on different age levels of prevalence and DALYs to provide a targeted analysis and point out the difference of ASD burden among different regions in Japan. From the comparison between stages we found that population prevalence with DALYs increased over time, both overall and by gender. Based on the difference contrasts of Age-cohort effect, we found that over time to 2021, the overall prevalence in Japan exceeded 1500 per 100000 population and DALYs also approached 350 per 100000 population. Among these, the male prevalence is nearly 2500 per 100000 population and DALYs also exceeds 400 per 100000 population, which far exceeds the overall ASD disease burden level in Japan, while the female prevalence is 1000 per 100000 population and DALYs does not exceed 200 per 100000 population (**Figures 6A, B**). However, based on the differential comparison of age-period effect, we found that after structured age, the prevalence and DALYs showed a negative correlation with age, and gradually decreased in both men and women, but the prevalence and DALYs were still much greater than that in women, for example male 2169.72(95%CI:1824.88, 2557.74) vs female 925.52(95%CI:776.29, 1099.33) per 100000 population/male 400.93(95%CI:279.32, 561.28) vs female 169.66(95%CI: 117.37, 234.72) per 100000 population of 50–54 years old people prevalence/DALYs in 2017–2021 and (**Figures 6C, D**). The differential comparison of the effects of period-cohort is consistent with the results of the former two (**Figures 6E, F**). In conclusion, we found that in the APC analysis, the overall disease burden of ASD in Japan increased with the period, showing a trend of young age, and the disease burden of men was much greater than that of women.

### 3.4 Comparison of disease burden in 47 Japanese regions based on AAPC and ASR

**Figure 7:**
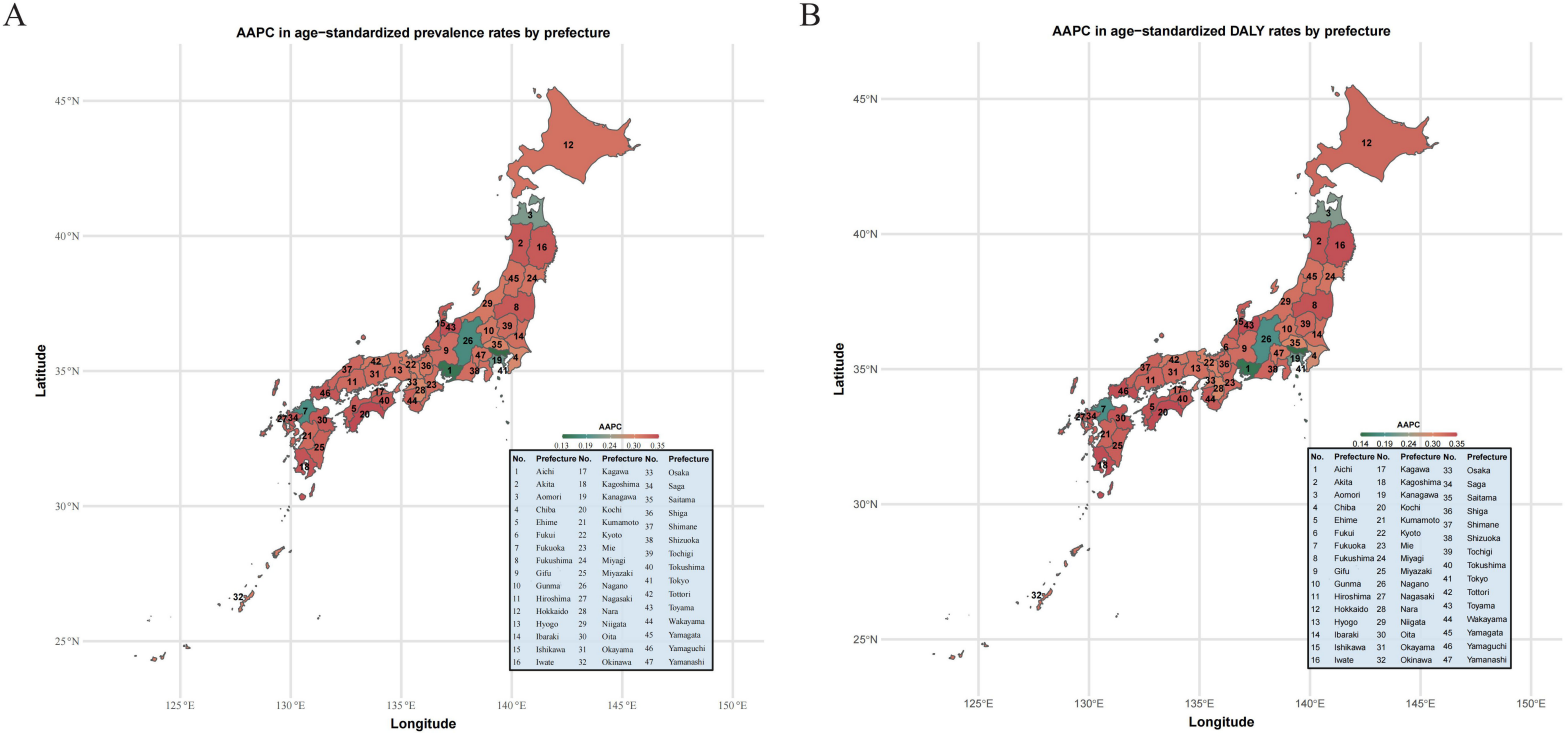
**(A)** AAPC in age−standardized prevalence rates by prefecture. **(B)** AAPC in age−standardized DALY rates by prefecture.

We first heat map the Japanese map against the AAPC values of the different administrative regions of Japan, applied the color difference to represent the level of AAPC, and ranked the 47 administrative regions according to the level of AAPC. The results suggested that the top three administrative regions with the highest AAPC were Aichi, Akita and Aomori, indicating that the disease burden of ASD was most significant and should be paid attention to (**Figure 7**). For a detailed description of the disease burden in these 47 regions, we drew calibration curves by adjusting for the effects of time, period, and cohort. Based on the disease characteristics of ASD, specific age groups (0–4 years) and specific periods (1988-1992) were calibrated and displayed with change curves (**Supplementary Figures S2–S11**).

### 3.5 Decomposition analysis

**Figure 8:**
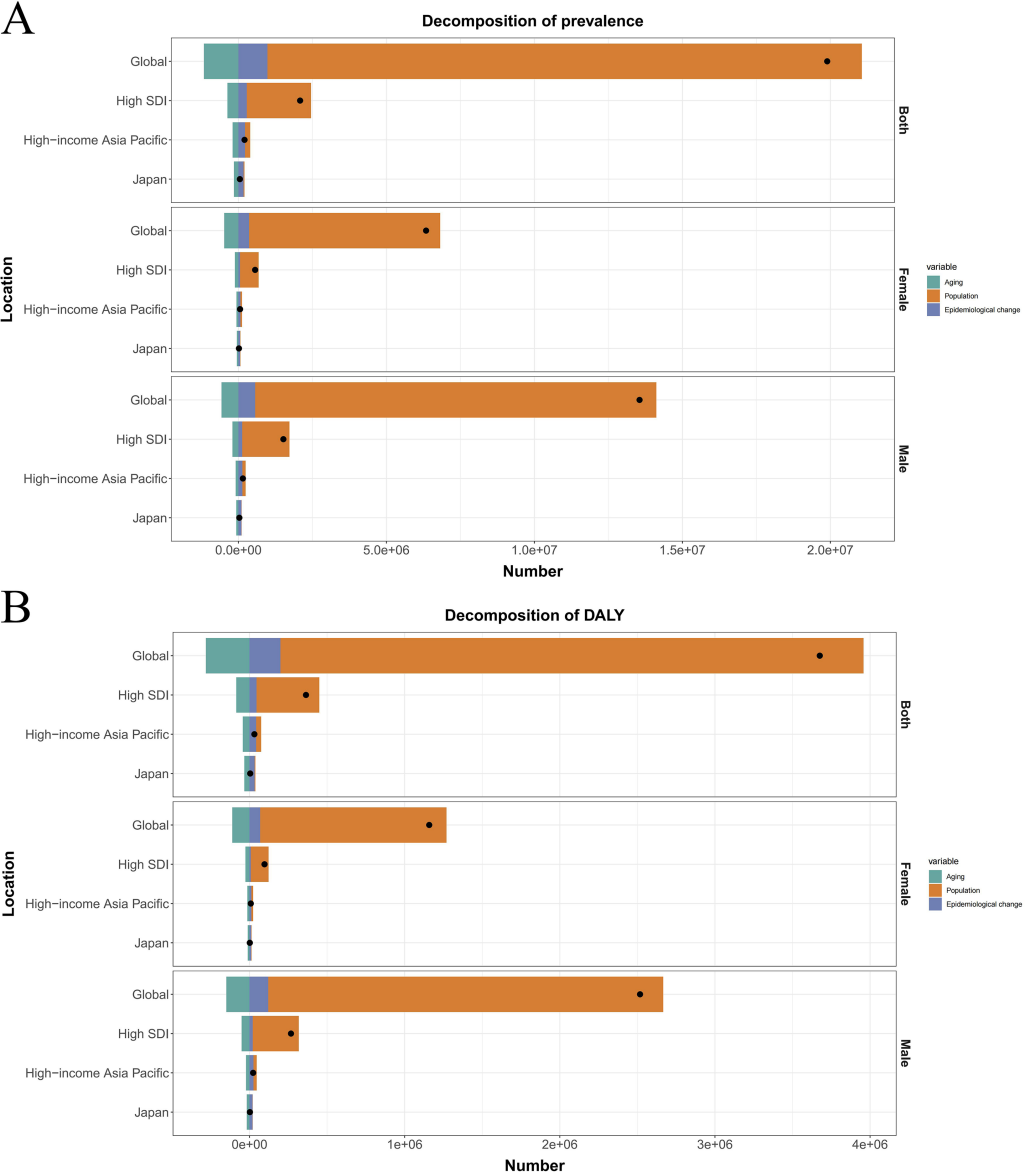
**(A)** Decomposition of prevalence into aging, population and epidemiological change by different regions. **(B)** Decomposition of DALYs into aging, population and epidemiological change by different regions.

We conducted a decomposition analysis to quantify contributions of three drivers to ASD burden changes between 1992 and 2021: Population growth (demographic expansion), Population aging (changing age structure), Epidemiological changes (disorder-specific risk factor prevalence) (**Figure 8**).

Globally, population growth was the dominant driver of increased ASD burden, accounting for 100.92% of the net prevalence change (+20,077,209 cases) and 102.26% of DALYs increase (+3,758,723 DALYs). This pattern held in high-SDI regions where population growth explained 104.32% of prevalence growth (+2,174,457 cases) and 110.90% of DALYs increase (+403,588 DALYs). Counteracting effects were observed globally: population aging reduced prevalence by 5.85% (-1,163,326 cases) and DALYs by 7.66% (-281,711 DALYs), while epidemiological changes increased prevalence by 4.92% (+979,662 cases) and DALYs by 5.40% (+198,639 DALYs). Similar trends occurred in high-SDI regions where aging reduced prevalence by 17.72% and epidemiological changes increased it by 13.40%. Japan demonstrated a divergent pattern. Population growth contributed moderately to prevalence (+26,831 cases, 55.02%) and DALYs (+4,986 DALYs, 112.50%). Crucially, population aging exerted substantial protective effects, reducing expected prevalence by 310.13% (-151,238 cases) and DALYs by 741.51% (-32,862 DALYs). Conversely, epidemiological changes drove the largest burden increases, accounting for 355.11% of net prevalence change (+173,173 cases) and 729.01% of DALYs change (+32,308 DALYs).

### 3.6 Prediction analysis

**Figure 9:**
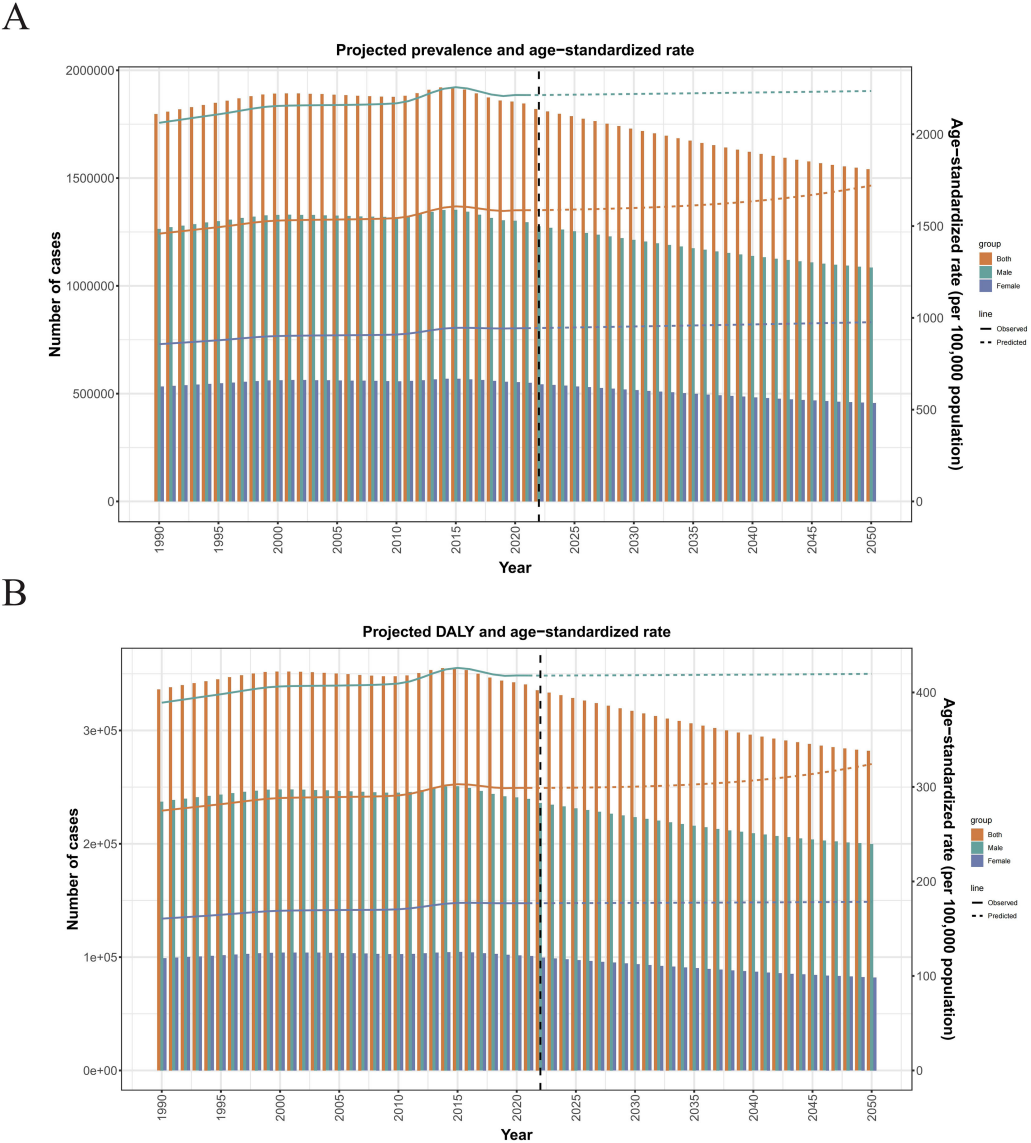
**(A)** Projected prevalence and ASPR. **(B)** Projected DALYs and ASDAR.

Moreover, the prediction results of the disease burden of ASD in Japan from 2022 to 2050 indicate that the ARIMA model predicted that disease burden prevalence and DALYs decrease annually, while their ASR increases continuously. By 2050, The prevalence prediction value of Japanese ASD in the ARIMA model will be calculated from 1797373.45551684 (1778254.63676399, 1816492.2742697) reduced to 1541547.43833842 (452394.414619911, 2630700.46205692), However, the predicted value of ASPR will be changed from 1458.68139124526 (1443.34004784751, 1474.02273464301) increased to 1720.90869986909 (482.783942768444, 2959.03345696973) (**Figure 9A**). Also, for the DALYs, In the prediction model from 336217.5175970309 in 1990 (328564.755586865, 343870.279607753) reduced to 281936.436409579 (63479.18170497, 500393.691114189), At the same time ASDAR predicted from 274.91919684919635 (268.74443821172, 281.084931627551) upgraded to 324.228788001257 (67.6272352112524, 580.830340791262) (**Figure 9B**).

## 4 Discussion

This study comprehensively analyzes the burden of ASD in Japan from 1990 to 2021 and projects trends until 2050 using data from the Global Burden of Disease (GBD) study. Our findings confirm a significant and sustained increase in the prevalence and overall disease burden of ASD in Japan over the past three decades, a trend consistent with reports globally and within other high Socio-demographic Index (SDI) regions (21, 22).

### 4.1 Rising prevalence and diagnostic trends

Multiple epidemiological studies within Japan align with our observed increase in ASD prevalence. Research in Yokohama reported a cumulative incidence of 16.2 per 10,000 and a prevalence of 21.1 per 10,000 for children born in 1988 (3). Similarly, studies in Nagoya and Fukushima-ken documented prevalence rates significantly higher than historical estimates (0.13% and 4.96 per 10,000, respectively), with notable urban-rural gradients (13, 26). This upward trajectory is largely attributable to enhanced detection methods, broader diagnostic practices aligned with evolving criteria (DSM-5-TR, ICD-11), and increased societal and professional awareness. These factors have improved identification, particularly of individuals with milder phenotypes or without co-occurring intellectual disability, who were previously underdiagnosed (27). Furthermore, reduced stigma surrounding neurodevelopmental conditions encourages more families to seek assessment (28).

### 4.2 Disease burden and societal implications

The escalating prevalence directly translates to a growing societal burden, demanding substantial expansion of specialized educational, healthcare, and support services (29). Our analysis of Age-Standardized Rates (ASR) and Average Annual Percentage Change (AAPC) underscores the severity of the current situation in Japan relative to global, high-SDI, high-income, and other Japanese administrative region averages. The high prevalence of co-existing neurodevelopmental and psychiatric conditions (e.g., reported co-occurrence rates around 3.22% for other NDDs alongside ASD (28)) further amplifies the complexity and resource intensity of required support, necessitating comprehensive, multi-disciplinary care strategies (30). Critically, our projections indicate this burden will continue to rise steadily through 2050, demanding urgent long-term national planning for service provision and workforce training.

### 4.3 Gender disparities and diagnostic patterns

A pronounced male predominance in ASD prevalence was consistently observed in our data, mirroring global patterns (approximately 3:1 male-to-female ratio) (31). While biological factors likely contribute significantly to this disparity, evolving diagnostic practices and awareness may also influence detection rates differentially by gender. Females, particularly those without intellectual impairment, may present differently or employ more effective masking strategies, potentially leading to under-identification (7, 10). Our data also reflects a declining age of diagnosis over the study period, indicating improved early detection efforts (28).

### 4.4 Regional variations and potential influences

Consistent with earlier reports (27), our analysis identified regional heterogeneity within Japan, with higher ASD burden indices often observed in more densely populated or complex urban environments. This pattern may reflect disparities in access to diagnostic services, specialist availability, or heightened parental awareness in urban centers (32), rather than solely indicating environmental etiology. While the search for etiological factors continues, encompassing genetic predispositions and potential environmental influences (e.g., prenatal/perinatal factors implicated in some studies (13, 32)), it is crucial to note that large-scale epidemiological evidence refutes a causal link between vaccines and ASD incidence. The withdrawal of the MMR vaccine in Japan did not alter the upward trajectory of ASD diagnoses (26).

### 4.5 Projections and global context

Our projection models, extending the robust GBD methodology (21), indicate a persistent rise in ASD prevalence and associated disability burden in Japan through 2050. This trend aligns with projections for other high-income nations, reflecting the ongoing impact of diagnostic broadening, sustained awareness efforts, and the aging of existing prevalent cases (21, 25). While the GBD 2021 Autism Collaborators highlight the global increase in ASD burden (22), our Japan-specific analysis reveals a burden profile exceeding the averages for high-SDI and high-income regions. This emphasizes the acute and growing challenge ASD presents within the Japanese healthcare and social support systems.

## 5 Conclusion

The rising burden of ASD in Japan, confirmed by our analysis of GBD data and projected to increase through 2050, results from a complex interplay of improved ascertainment, evolving diagnostic criteria, heightened awareness, and true increases in prevalence. The significant male predominance and regional variations require further investigation into biological mechanisms and equitable access to services. The projected increase necessitates immediate and sustained investment in early identification programs, evidence-based interventions, lifespan support services, and targeted resource allocation, particularly in regions showing higher burden. Future research must prioritize understanding the drivers of the increase, refining early detection across genders, evaluating the long-term effectiveness of support services, and developing strategies to mitigate the projected burden on individuals, families, and society.

## Data Availability

The original contributions presented in the study are included in the article/**Supplementary Material**. Further inquiries can be directed to the corresponding authors.
