# Supplementary figures and images for: Burden of autism spectrum disorder in Japan from 1992 to 2021 and its prediction until 2050: results from the GBD study
Source: Front Psychiatry. 2025 Jul 8;16:1619085. doi: 10.3389/fpsyt.2025.1619085 (PMC12279874; doi:10.3389/fpsyt.2025.1619085)

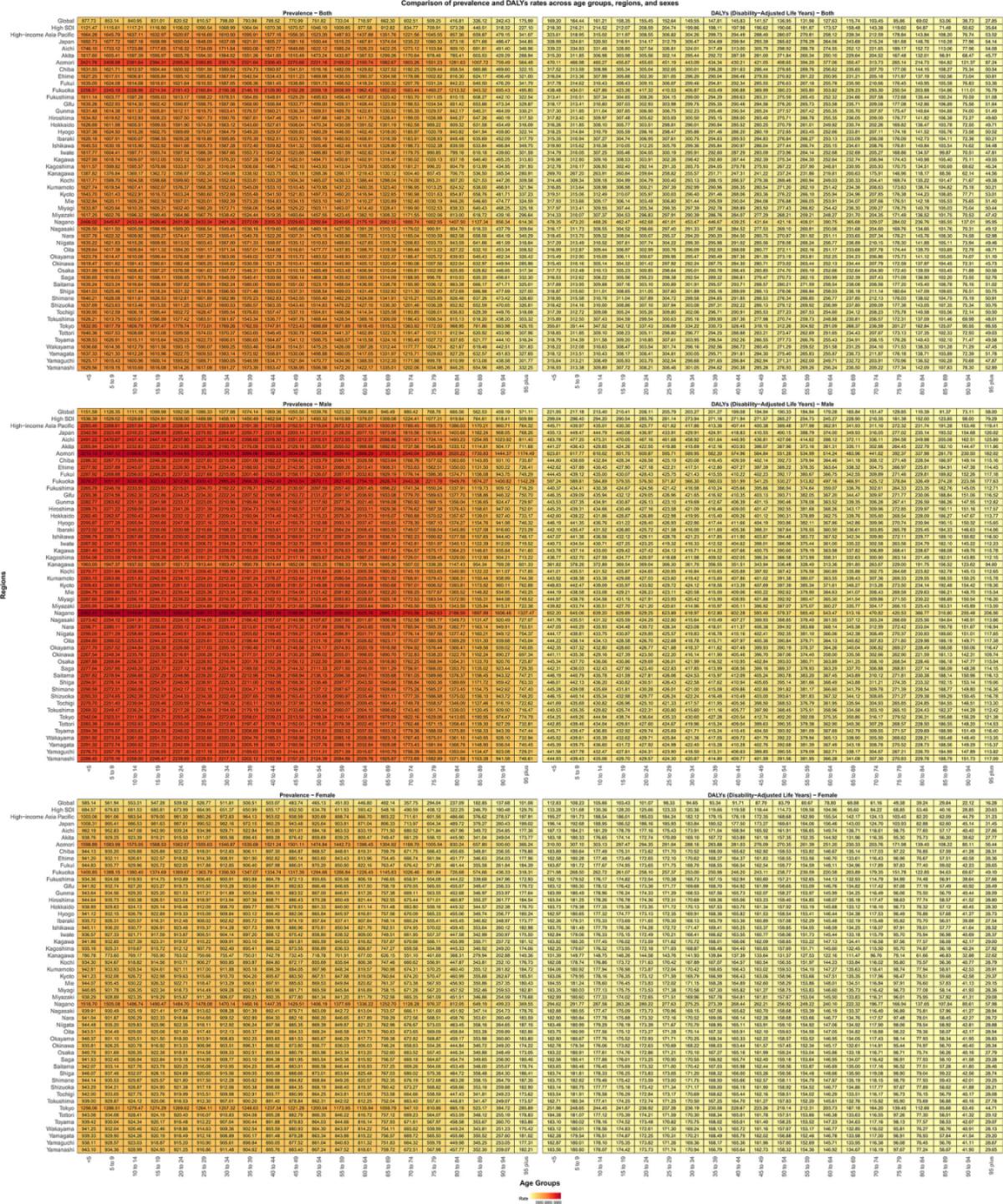

Supplement: Supplementary file 1 [file Image1.jpeg]

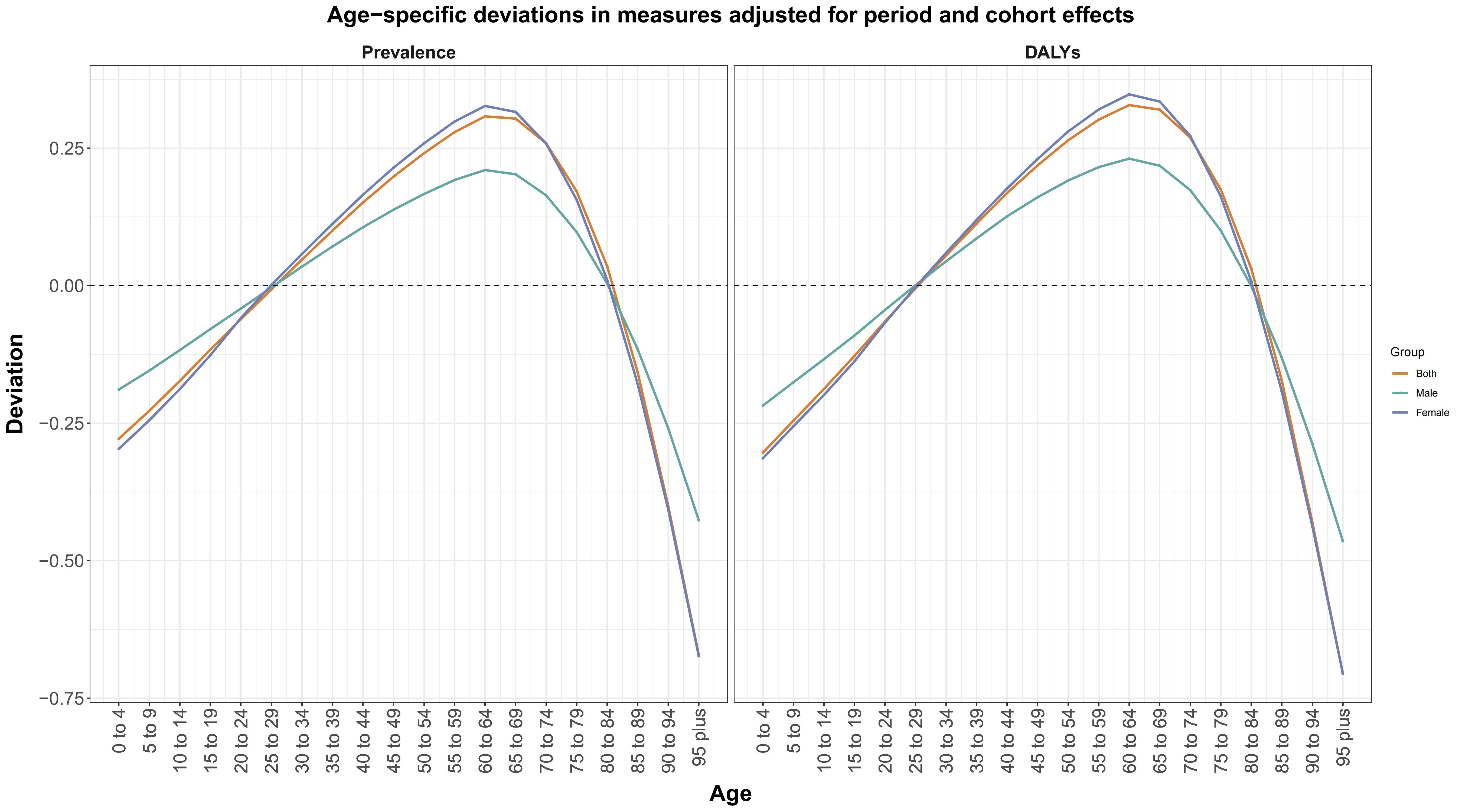

Supplement: Supplementary file 2 [file Image2.jpeg]

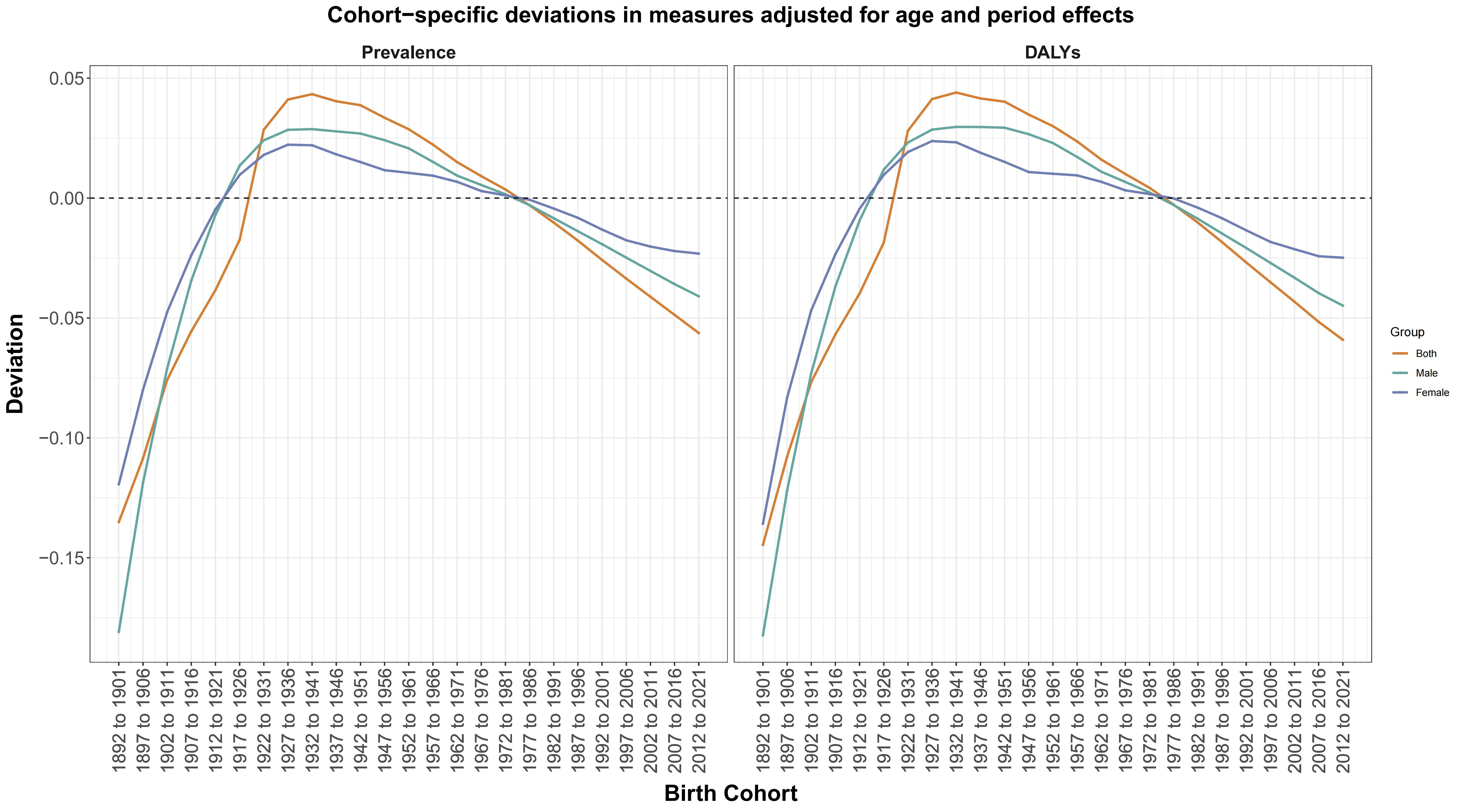

Supplement: Supplementary file 3 [file Image3.jpeg]

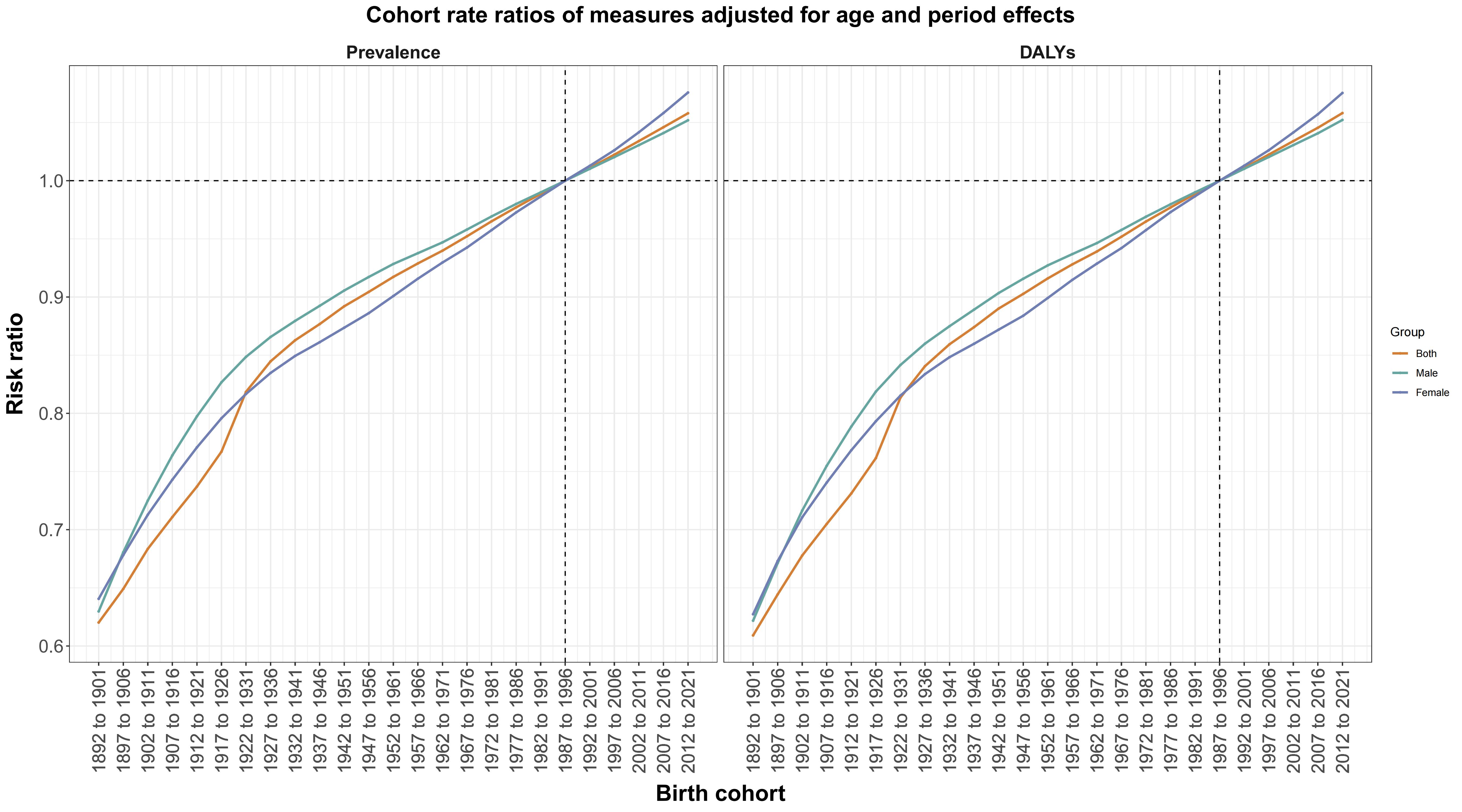

Supplement: Supplementary file 4 [file Image4.jpeg]

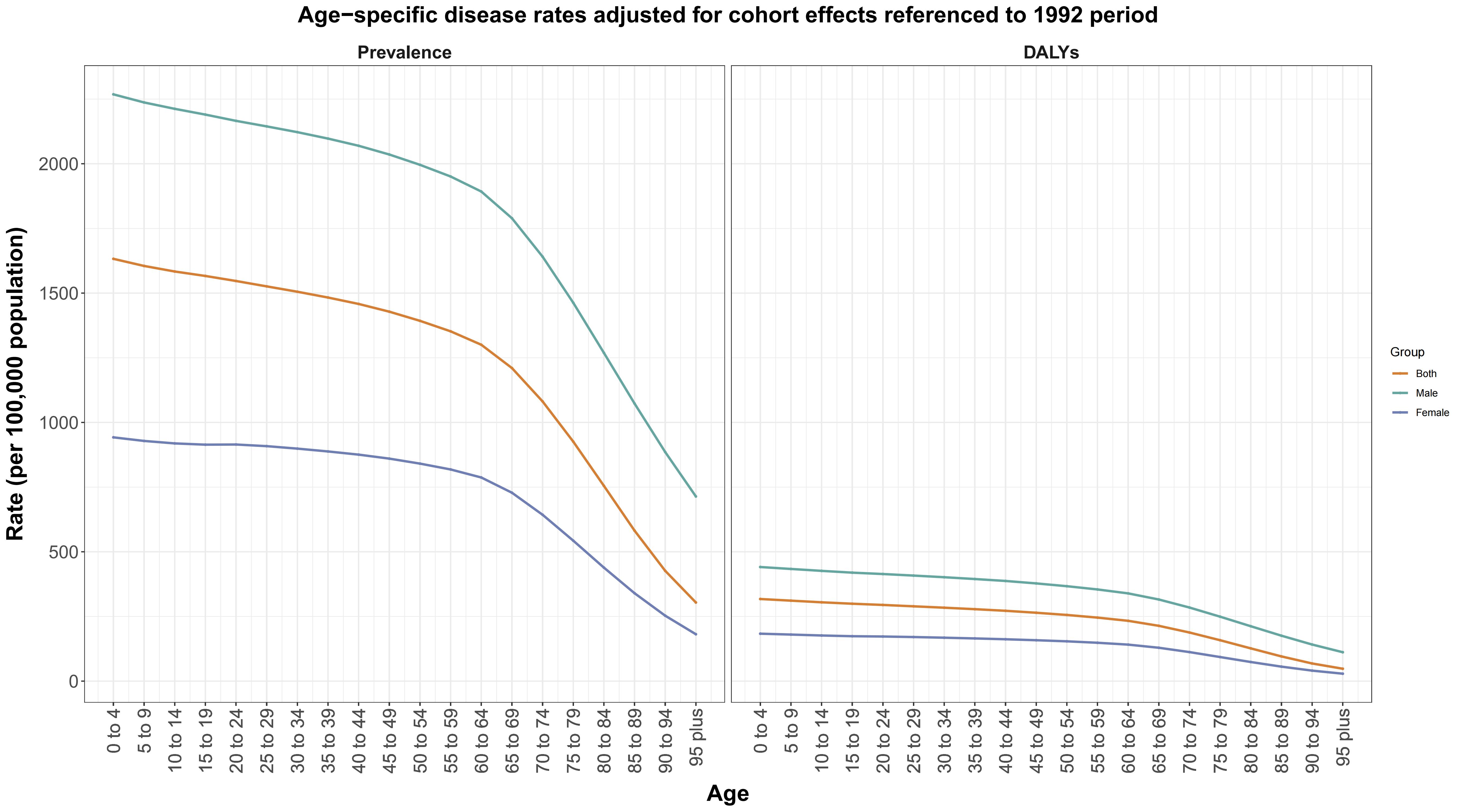

Supplement: Supplementary file 5 [file Image5.jpeg]

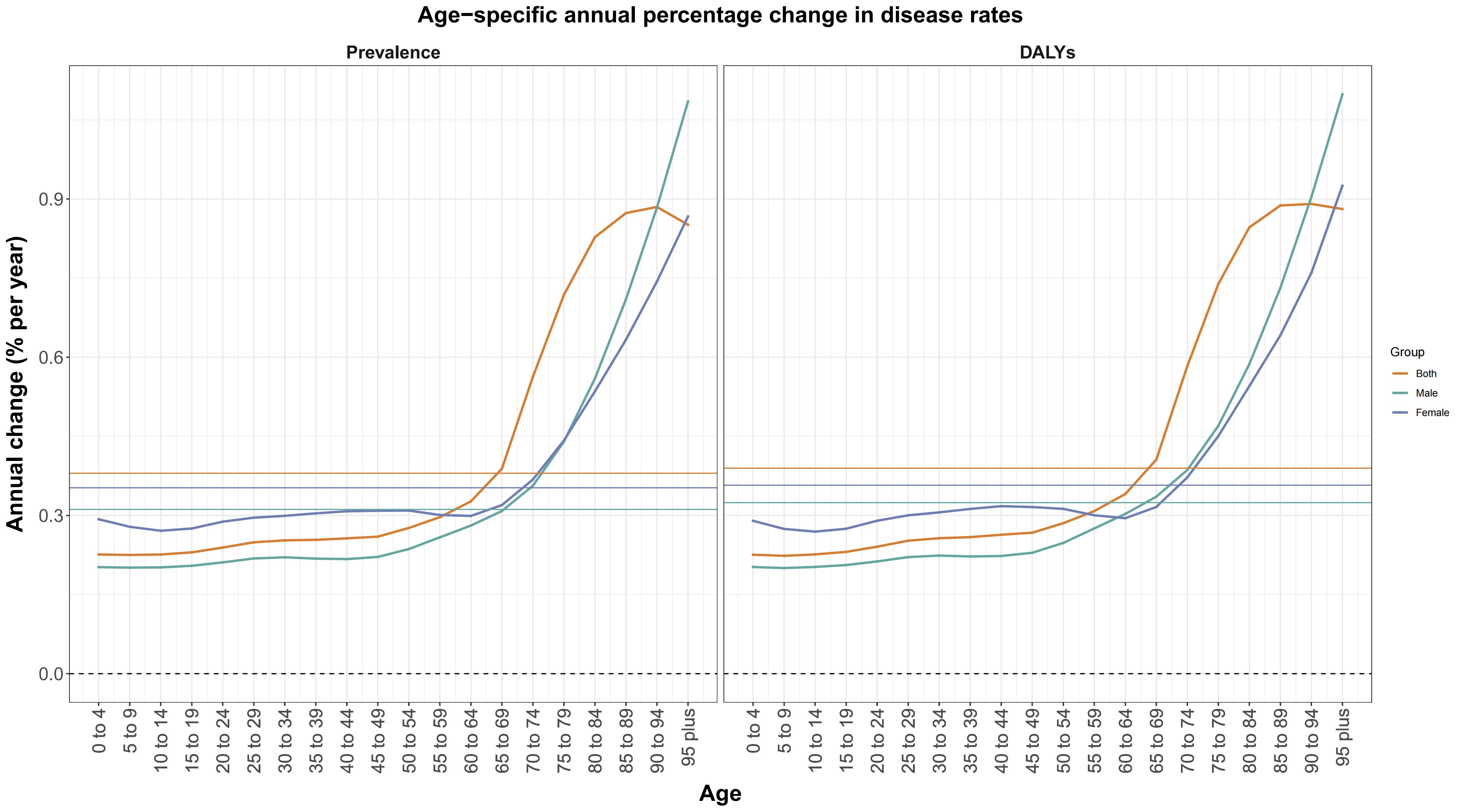

Supplement: Supplementary file 6 [file Image6.jpeg]

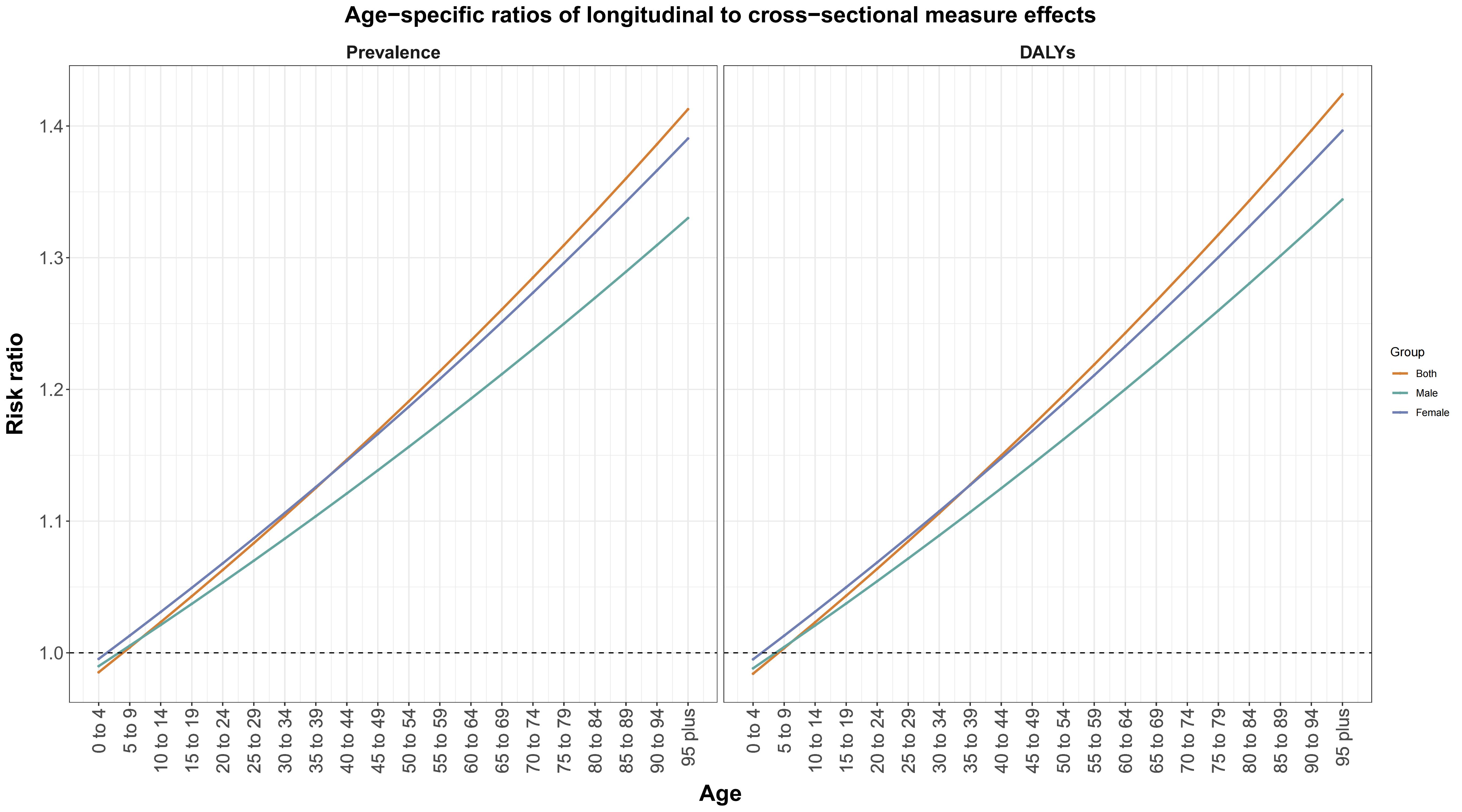

Supplement: Supplementary file 7 [file Image7.jpeg]

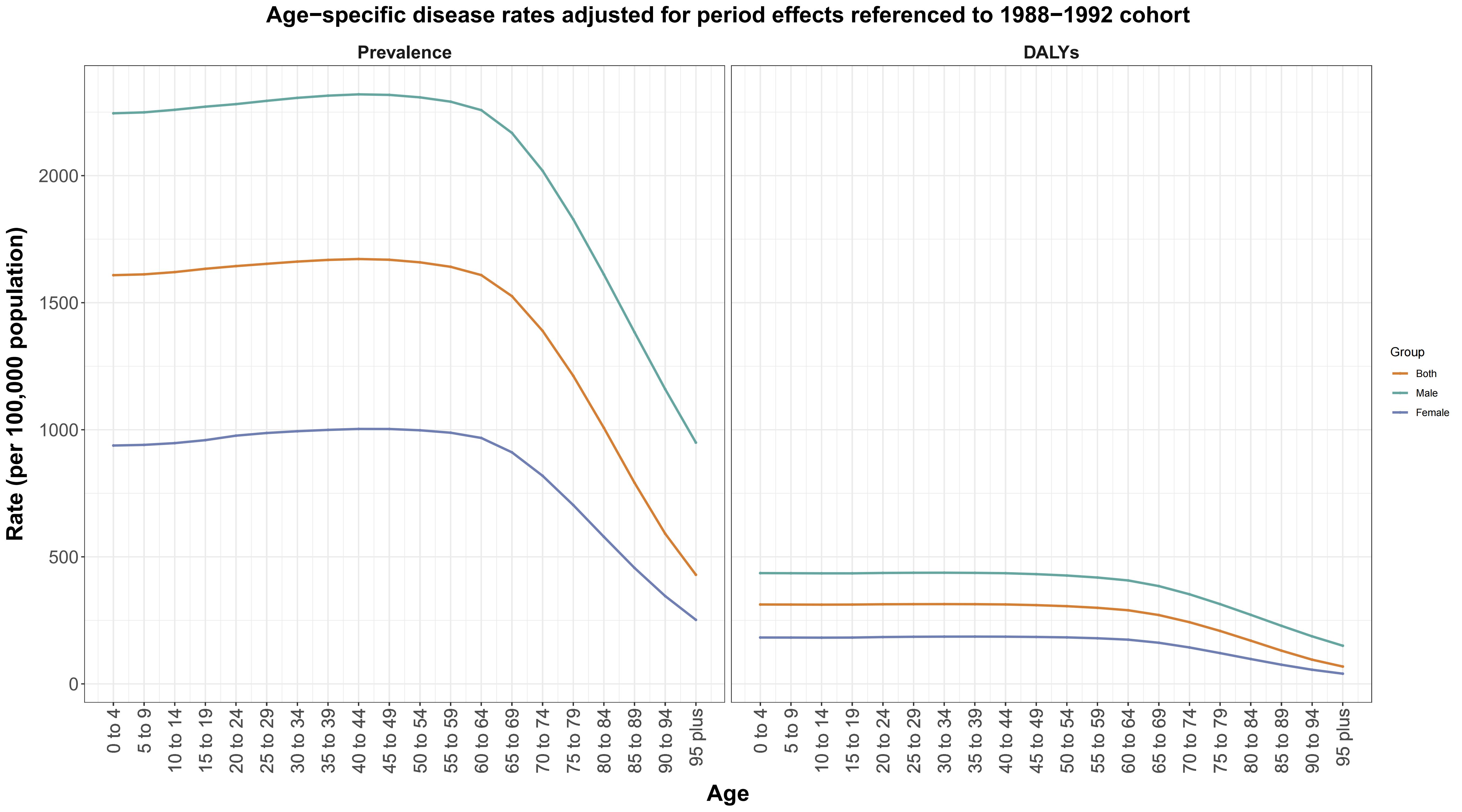

Supplement: Supplementary file 8 [file Image8.jpeg]

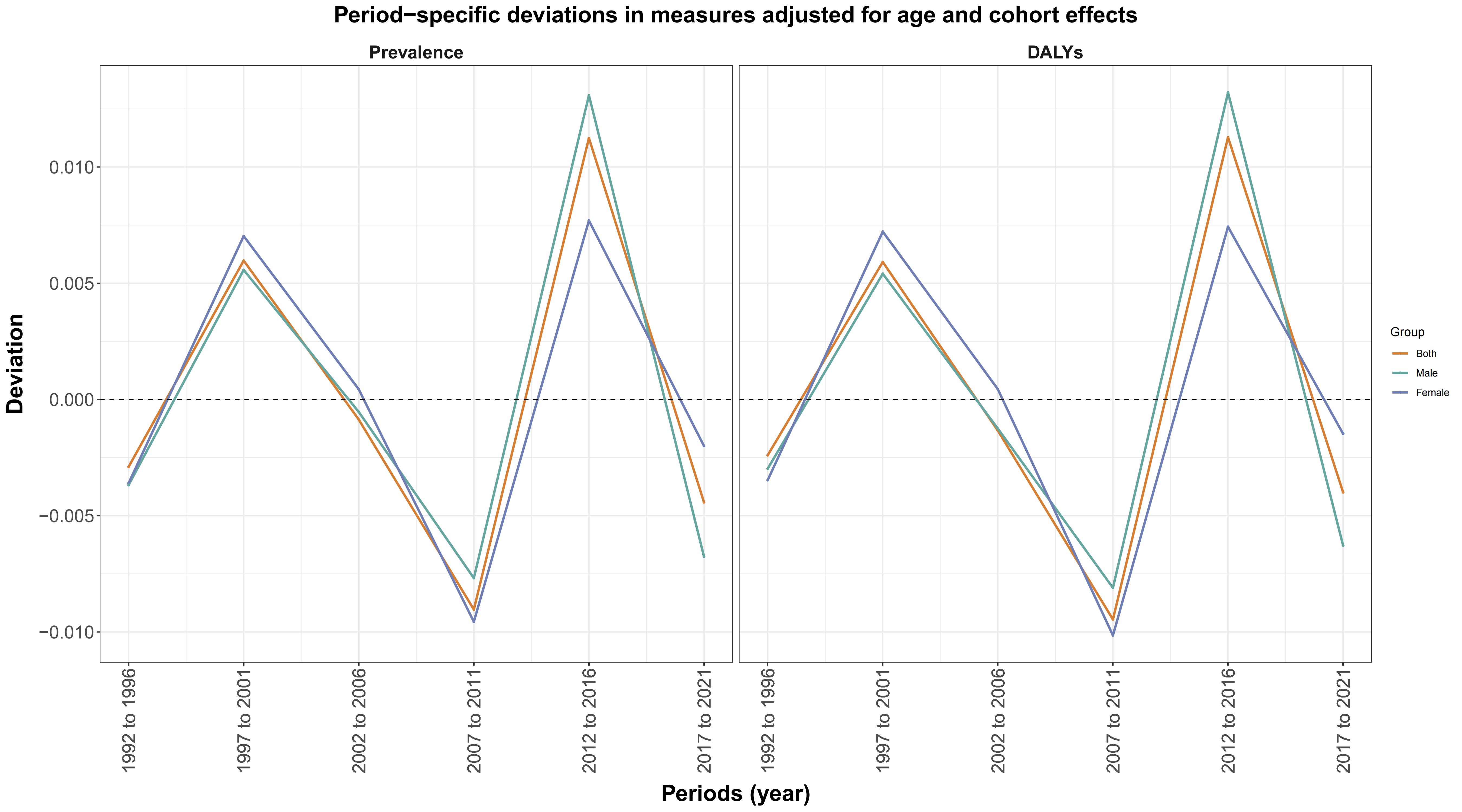

Supplement: Supplementary file 9 [file Image9.jpeg]

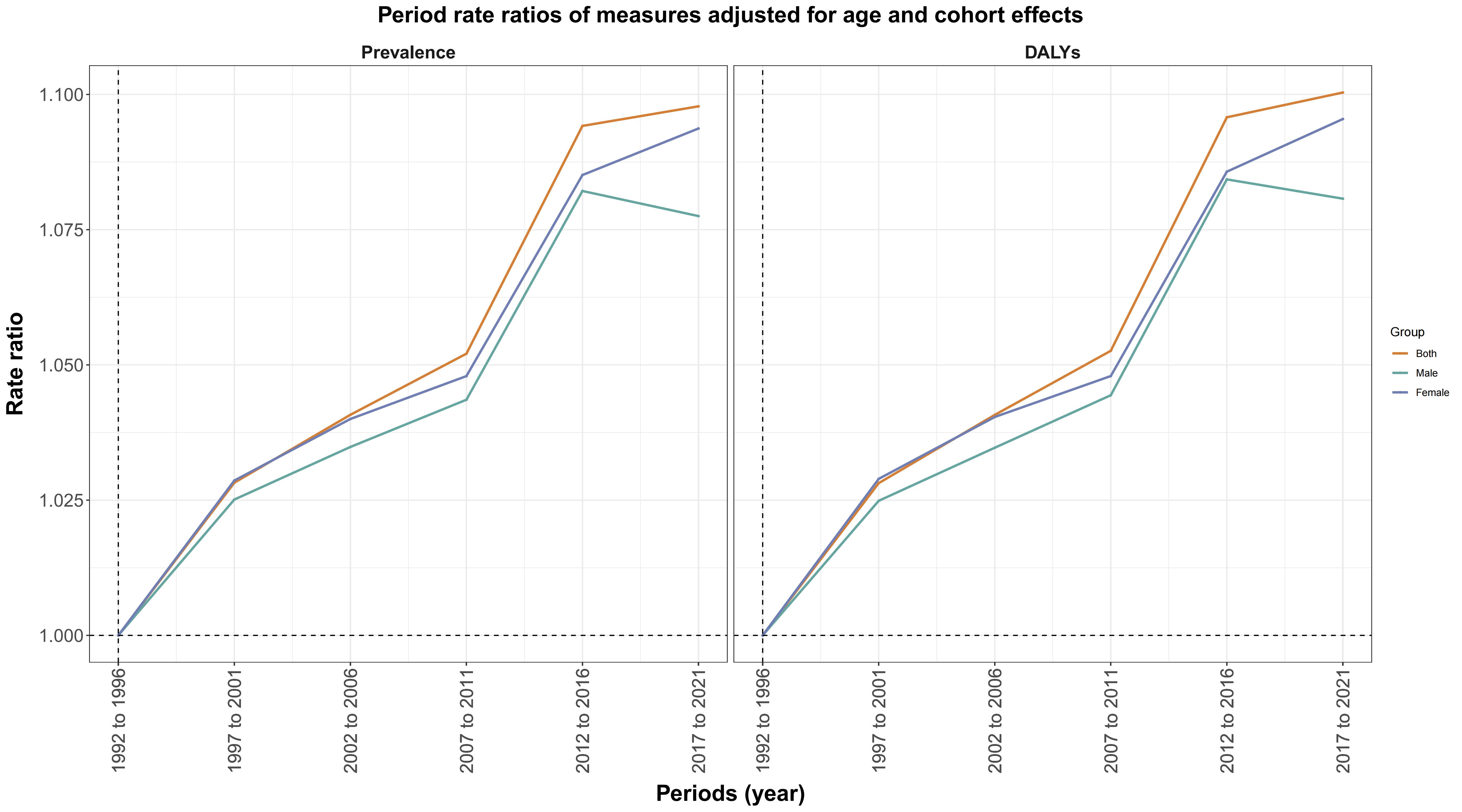

Supplement: Supplementary file 10 [file Image10.jpeg]

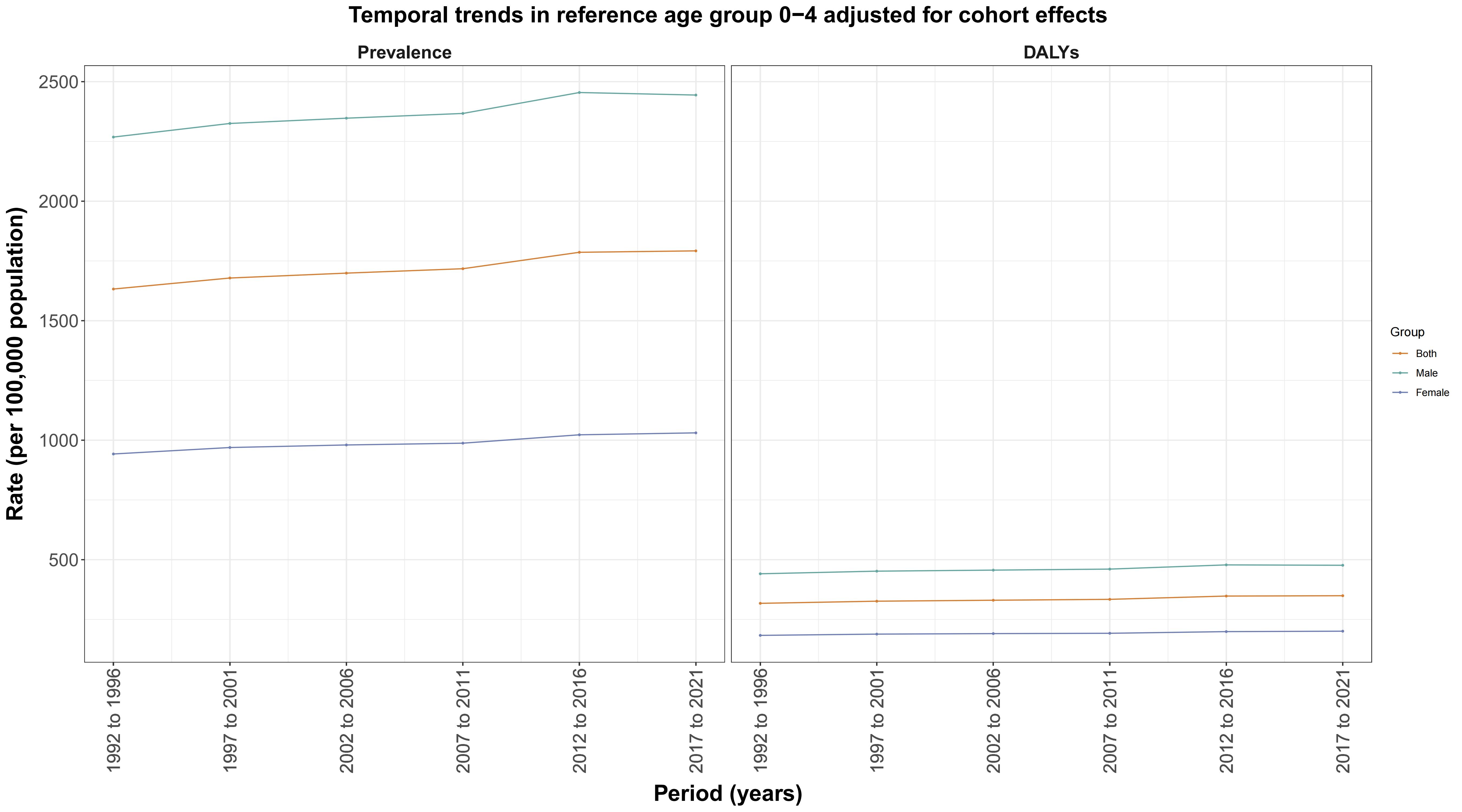

Supplement: Supplementary file 11 [file Image11.jpeg]
